# Rare Case of Reversible Pulmonary Arterial Hypertension Secondary to Cyclophosphamide and Doxorubicin Chemotherapy

**DOI:** 10.7759/cureus.26207

**Published:** 2022-06-22

**Authors:** Aneeqa Javed, Yelizaveta Medina, Amber Bux, Syeda Sahra, Geurys Rojas-Marte

**Affiliations:** 1 Internal Medicine, Northwell Health, Staten Island, USA; 2 Cardiology, Northwell Health, Staten Island, USA

**Keywords:** reversible pulmonary hypertension, chemotherapy, doxorubicin, cyclophosphamide, pulmonary hypertension

## Abstract

Pulmonary arterial hypertension (PAH), characterized as a resting mean pulmonary artery pressure greater than 25 mmHg, is due to the narrowing of the pulmonary arteries, which can be idiopathic, inherited, or drug-related. Alkylating agents, including cyclophosphamide, are a risk factor for developing the pulmonary veno-occlusive disease. Drug-induced PAH is extremely rare. A 59-year-old female with newly diagnosed invasive ductal carcinoma of the right breast and high-grade ductal carcinoma in situ of the left breast was initiated treatment with doxorubicin and cyclophosphamide. About one week after receiving the first cycle, the patient developed worsening lower extremity edema and shortness of breath. She was then hospitalized, and a transthoracic echocardiogram and coronary angiogram revealed PAH. The team then changed the breast cancer treatment regimen to Taxol and carboplatin, and PAH was resolved in a follow-up echocardiogram after five months. This report has described the first case of PAH directly related to cyclophosphamide and doxorubicin. It is imperative to promptly recognize this rare but important side-effect as early diagnosis and response can potentially reverse the disease progression.

## Introduction

Pulmonary arterial hypertension (PAH) is characterized as a resting mean pulmonary artery pressure >25mmHg. It happens when the pressure in the blood vessels leading from the heart to the lungs is elevated. The World Health Organization (WHO) divides pulmonary hypertension into five categories. Group one is known as PAH due to narrowing of the pulmonary arteries, which can be idiopathic, hereditary, or drug-related. Group two is due to left heart systolic and diastolic dysfunction or valvular disease. Group three is secondary to pulmonary causes such as chronic obstructive pulmonary disease (COPD) or hypoxemia. Group four is due to obstruction of the pulmonary circulation such as malignancy, benign mass, or inflammation. Lastly, group five's etiology is unclear/multifactorial such as secondary to hematological diseases or metabolic disorders [1].

Although pulmonary hypertension can be categorized into five different types, the principal indicator of all morphologies of pulmonary hypertension is increasing exercise dyspnea. It is usually escorted by tiredness and fatigue. Due to the non-specificity of associated symptoms, establishing a diagnosis can be delayed for months to years. Due to the delay in recognition, progression of the disease leads to eventually dyspnea on bending down and syncope mainly after or during exertion. Frequent syncopal episodes on slight exertion are associated with higher mortality and cardiac decompensation. The rise in right heart cardiac pressures is associated with venous congestion, abdominal ascites, and lower extremity edema [2].

Pulmonary hypertension continues to be an untreatable ailment. The gold standard to diagnose pulmonary hypertension is right heart catheterization. In patients with idiopathic, heritable, or drug-related PAH, right heart catheterization is supplemented by vasoreactivity testing to recognize the responders who may benefit from medication with high-dose calcium antagonists. However, treatment with calcium works out in fewer than 5% of patients with PAH and must be monitored closely once treatment is initiated. The goal of the treatment is to slow down the progression of the disease and stabilize the patient without signs of right heart failure. Treatment in newly diagnosed PAH with low or intermediate risk can be initiated on a combination compromising an endothelin receptor antagonist (ERA) with a phosphodiesterase-5 (PDE5) inhibitor or a soluble guanylate cyclase (sGC) stimulator. We can use a three-layered blend of an ERA, a PDE5 inhibitor, an sGC stimulator, and an intravenously administered prostacyclin analog for the high-risk patient category. If treatment response remains inadequate, patients should be evaluated for lung transplant without delay [2].

In conclusion, pulmonary hypertension is most associated with left-sided heart failure or secondary to lung diseases. Other causes are not as commonly seen and thought to be rare. As such, we present this unique case of chemotherapy-induced pulmonary hypertension.

## Case presentation

A 59-year-old female with newly diagnosed invasive ductal carcinoma of the right breast and high-grade ductal carcinoma in situ of the left breast was initiated treatment with doxorubicin and cyclophosphamide. About one week after receiving the first cycle, the patient developed worsening lower extremity edema and shortness of breath. She was then hospitalized, and a transthoracic echocardiogram (TTE) revealed moderately reduced right ventricular function, enlarged right ventricle with severe pulmonary hypertension, and estimated pulmonary artery systolic pressure of 79.5 mmHg, assuming a right atrial pressure of 10 mmHg as seen in Videos 1, 2.

**Video 1:** Transthoracic echocardiogram showing moderately reduced right ventricular function and enlarged right ventricle.

**Video 2:** Moderately reduced right ventricular function, enlarged right ventricle with severe pulmonary hypertension, estimated pulmonary artery systolic pressure of 79.5 mmHg, assuming a right atrial pressure of 10 mmHg based on echocardiography calculations.

Coronary angiogram revealed severe pulmonary hypertension without response to nitric oxide, mild to moderate elevation in left ventricular end-diastolic pressure, pulmonary capillary wedge pressure, and normal coronary anatomy (Videos 3, 4).

**Video 3:** Coronary angiogram revealed severe pulmonary hypertension without response to nitric oxide, mild to moderate elevation in left ventricular end-diastolic pressure, pulmonary capillary wedge pressure, and normal coronary anatomy

**Video 4:** Coronary angiogram revealed severe pulmonary hypertension without response to nitric oxide, mild to moderate elevation in left ventricular end-diastolic pressure, pulmonary capillary wedge pressure, and normal coronary anatomy

**Figure 1:**
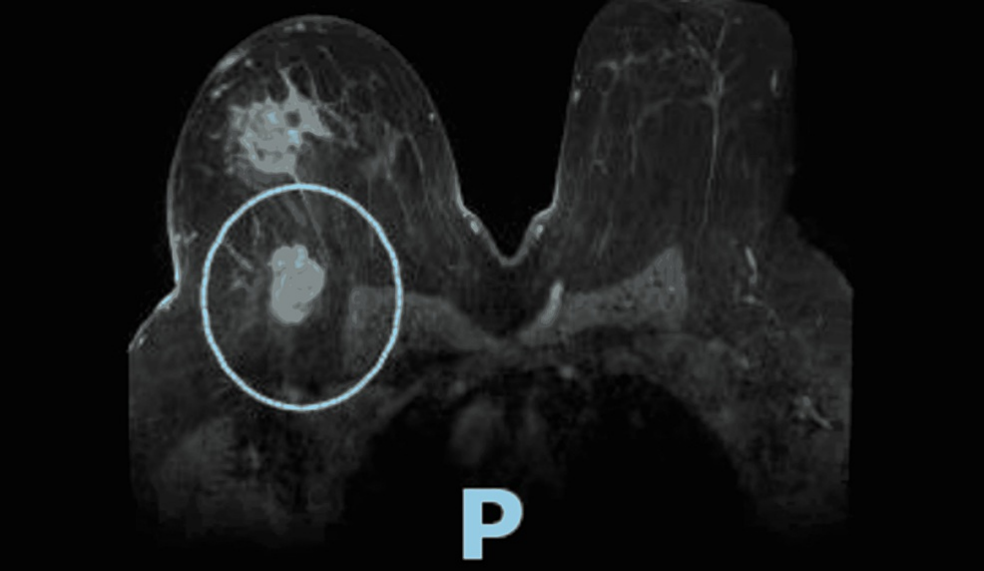
Baseline size of right breast carcinoma and surrounding satellite nodules.

**Figure 2:**
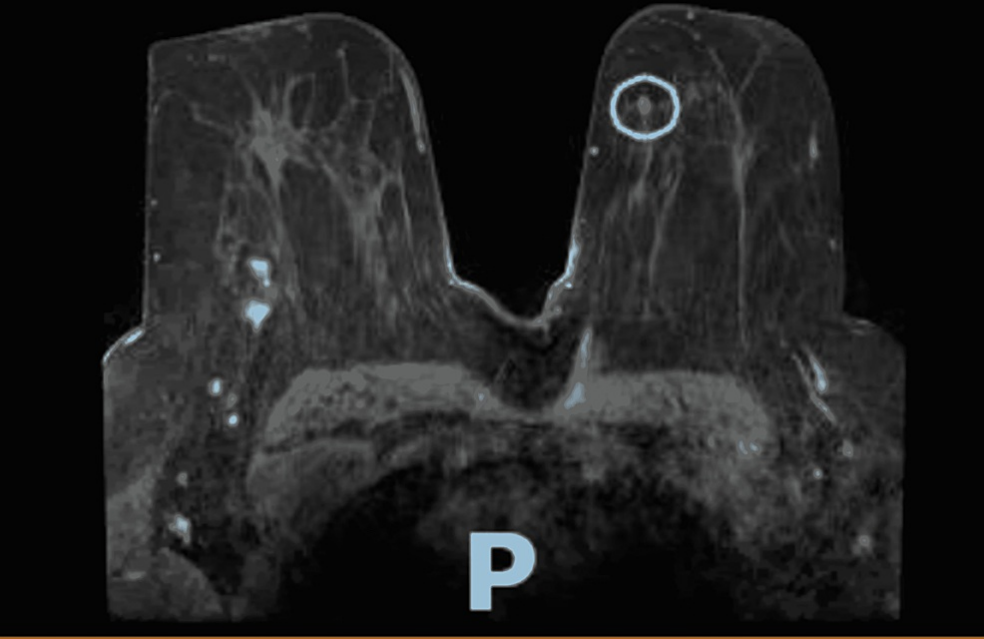
Breast MRI revealed interval decrease in size of right breast carcinoma and resolution of surrounding satellite nodules.

TTE obtained about one month before initiating chemotherapy revealed normal right ventricular size and function, mild pulmonary hypertension, estimated pulmonary artery systolic pressure of 44.8 mmHg, assuming right atrial pressure of 3 mmHg. The team then changed the breast cancer treatment regimen to Taxol and carboplatin, of which the patient completed 12 cycles; complications were noted to be fatigue and pancytopenia. Breast MRI revealed interval decrease in size of right breast carcinoma and resolution of surrounding satellite nodules (comparison of Figures 1, 2) showing the pre and post chemotherapy effects on size of lesion.

Five months after the patient was diagnosed with pulmonary hypertension, repeat TTE was obtained and revealed normal right ventricular size, mildly reduced right ventricular systolic function, no pulmonary hypertension with pulmonary artery systolic pressure of 20.7 mmHg, normal filling pressures, and moderately reduced left ventricular systolic function.

## Discussion

The WHO divides pulmonary hypertension into five subgroups. Group 1 pulmonary hypertension, also known as PAH, is due to the narrowing of pulmonary arteries, which can be idiopathic or secondary to genetic causes, certain medical conditions, or various drugs [3,4]. Drug-induced PAH is extremely rare, with only 10.5% of cases reported in large registry series [5].

Several chemotherapeutic agents have been associated with pulmonary complications, including pulmonary fibrosis, pulmonary hypertension, and interstitial pneumonitis. A systematic review of the role of alkylating agents in the development of pulmonary hypertension published in 2015 established that alkylating agents, including cyclophosphamide, are a risk factor for the development of pulmonary veno-occlusive disease (PVOD), and exposure to cyclophosphamide leads to pulmonary venous remodeling in experimental models which, in turn, develops pulmonary hypertension [6]. PVOD is extremely rare, with its incidence being 0.1 to 0.2 cases per million every year [7], and clinically very difficult to differentiate from PAH. The gold standard for diagnosis of PAH is right heart catheterization. Our patient developed PAH confirmed by right heart catheterization after only one cycle of cyclophosphamide and doxorubicin.

The metabolism of cyclophosphamide in the lung is partly responsible for its pulmonary toxicity. Data suggest that cyclophosphamide and its metabolites cause peroxidative injury of the membrane lipids [8]. Doxorubicin is notorious for its cardiotoxicity hypothesized to be due to oxidative stress and cardiac mitochondrial damage leading to cardiomyopathy [9].

We have described the first case of PAH directly related to cyclophosphamide and doxorubicin. Since our patient received both drugs and both were discontinued when the patient developed PAH, we cannot establish which chemotherapeutic agent was responsible. However, there is more data on the pulmonary toxicity of cyclophosphamide than doxorubicin [10,11]. Interestingly, cyclophosphamide has been effectively used in the therapy of PAH in diseases akin to systemic lupus erythematosus and mixed connective tissue disease [12,13].

The uniqueness of our case stems from the fact that after discontinuation of the chemotherapeutic agents, our patient's pulmonary hypertension resolved, showing that this effect might be reversible. It is imperative to recognize this rare but important side-effect as early diagnosis and response can potentially reverse the disease progression.

## Conclusions

Pulmonary hypertension remains challenging to treat. The most common cause of pulmonary hypertension is left-sided heart failure, but it can occur secondary to chemotherapeutic drugs. Our patient developed pulmonary hypertension after one cycle of chemotherapy with doxorubicin and cyclophosphamide, confirmed with a right heart catheterization. It resolved after the discontinuation of these drugs. In conclusion, chemotherapeutic drugs are often the offending agent in causing pulmonary complications, and when using these treatment regimens, the potential side effects should be kept in mind.
